# Erratum: “Comparing the Iowa and Soochow gambling tasks in opiate users”

**DOI:** 10.3389/fpsyg.2013.00085

**Published:** 2013-02-26

**Authors:** Daniel J. Upton, Rebecca Kerestes, Julie C. Stout

**Affiliations:** School of Psychology and Psychiatry, Monash UniversityMelbourne, VIC, Australia

We would like to apply a correction to the pay-off distributions of the Soochow Gambling Task presented in Table 2 of the manuscript. Decks C and D do not yield a gain on every trial, which has been corrected with the addition of a “–” in columns representing decks C and D. Furthermore, the 5th and 10th trials for both decks C and D in the Soochow Gambling Task, are associated with a win of $5.25 and $3.25, respectively. This was previously not shown in the table and has been inserted.

**Table 2:** **The pay-off distributions of the Iowa Gambling Task and Soochow Gambling Task for the first 10 trials, adapted and modified from Ahn et al. (2008)**.

**IGT**	**A**	**B**	**C**	**D**	**SGT**	**A**	**B**	**C**	**D**
Expected value of 10 trials	−$2.50	−$2.50	$2.50	$2.50	Expected value of 5 trials	−$2.50	−$2.50	$2.50	$2.50
Gain on every trial	$1.00	$1.00	$0.50	$0.50	Gain on every trial	$1.00	$0.50	–	–
Loss on each trial					Loss on each trial				
Trial 1								−$1.00	−$0.50
Trial 2								−$1.00	−$0.50
Trial 3								−$1.00	−$0.50
Trial 4								−$1.00	−$0.50
Trial 5						−$5.25	−$3.25	+$5.25	+$3.25
Trial 6	−$1.50		−$0.25					$1.00	−$0.50
Trial 7	−$2.00		−$0.75					$1.00	−$0.50
Trial 8	−$2.50		−$0.50					$1.00	−$0.50
Trial 9	−$3.00		−$0.50					$1.00	−$0.50
Trial 10	−$3.50	−$12.50	−$0.50	−$2.50		−$5.25	−$3.25	+$5.25	+$3.25
